# *VCP* expression decrease as a biomarker of preclinical and early clinical stages of Parkinson’s disease

**DOI:** 10.1038/s41598-020-57938-3

**Published:** 2020-01-21

**Authors:** Anelya Alieva, Margarita Rudenok, Elena Filatova, Alexey Karabanov, Olga Doronina, Kseniya Doronina, Anna Kolacheva, Mikhail Ugrumov, Sergey Illarioshkin, Petr Slominsky, Maria Shadrina

**Affiliations:** 1 Institute of Molecular Genetics, Russian Academy of Sciences, Moscow, Russia; 2 Research Centre of Neurology, Moscow, Russia; 3 Novosibirsk State Medical University, Novosibirsk, Russia; 4 Koltzov Institute of Developmental Biology, Russian Academy of Sciences, Moscow, Russia

**Keywords:** Gene expression, Parkinson's disease

## Abstract

Valosin-containing human protein (VCP) or p97 performs enzyme functions associated with the maintenance of protein homeostasis and control of protein quality. Disruption of its normal functioning might be associated with the development of Parkinson’s disease (PD). Tissues of mice with toxin-induced presymptomatic and early symptomatic stages of PD, as well as 52 treated and untreated patients with newly diagnosed PD and nine patients with a “predicted” form of PD, were investigated. Significant changes in *Vcp* gene expression were observed in almost all studied mouse tissues. A significant decrease in *VCP* expression specific for PD was also detected at both the late preclinical and the early clinical stages of PD in untreated patients. Thus, a decrease in *VCP* expression is important for changes in the function of the nervous system at early stages of PD. Analysis of changes in *VCP* expression in all patients with PD and in *Vcp* in the peripheral blood of mice used as models of PD revealed significant decreases in expression specific for PD. These data suggest that a decrease in the relative levels of *VCP* mRNA might serve as a biomarker for the development of pathology at the early clinical and preclinical stages of human PD.

## Introduction

Valosin-containing human protein p97, also known as VCP, belongs to the AAA + ATPase superfamily^1^. This enzyme performs many functions in cells, and maintenance of protein homeostasis and quality is among the most important^2,3^. VCP also acts in an ATP-dependent manner to separate aberrant proteins from large protein complexes or cellular structures, such as endoplasmic reticulum, membranes and mitochondria, for their subsequent transportation to proteasomes for degradation^4,5^. This enzyme is also involved in the release of defective products of mRNA translation that remain on ribosomes^6–8^.

Suppression of the expression of *VCP* can have a negative impact on the development of the nervous system. When antisense morpholino oligonucleotides were introduced to the *VCP* ortholog *cdc48* in zebrafish embryos, the development of affected embryos was accompanied by defects in neuronal outgrowth and neurodegeneration, and death. Accumulation of polyubiquitinated proteins was observed in the inner plexiform and ganglion cell layers of the brain, as well as in the diencephalon and mesencephalon. This indicates that the degradation of polyubiquitinated proteins by the ubiquitin–proteasome system was blocked in these brain structures. These abnormal phenotypes in zebrafish embryos were rescued by overexpression of *cdc48* or human *VCP*. Thus, VCP plays an important role in nervous system development by participating in the removal of aberrant proteins^9^.

Thus, a disturbance of normal VCP functioning can be associated with the development of various neurodegenerative disorders. Indeed, it has been shown that impairment of VCP functioning is involved in the accumulation of inclusions in expanding inclusion body myopathy with early-onset Paget disease and frontotemporal dementia^10–12^, and mutations in *VCP* can also be causative in Charcot–Marie–Tooth disease^13^ and amyotrophic lateral sclerosis^14^. Therefore, mutations in this gene in patients with a familial form of amyotrophic lateral sclerosis were revealed using whole-exome sequencing^15^ showing that mutations in the *VCP* gene can lead to impairments in autophagy in patients with this disorder^16^.

Moreover, it was shown that patients with neurodegenerative diseases and mutations in *VCP*^17,18^ demonstrated signs of parkinsonism including unilateral rigidity, tremor, and bradykinesia^14,19,20^. These data suggest that disruption of normal VCP functioning plays an important role in Parkinson’s disease (PD), which is the one of the most common neurodegenerative pathologies^21,22^.

PD is caused by impaired functioning of different neurotransmitter systems with a distinct predominance of dopaminergic (DAergic) system deficiencies^23,24^. The accumulation of misfolded proteins and the formation of protein inclusions, Levy’s bodies and neurites, primarily in the substantia nigra and striatum, are a characteristic histopathology feature of the disease^25^. As it was mentioned above, VCP plays an important role in the degradation of aberrant proteins, including misfolded proteins^3–5^. It is possible that the accumulation of such inclusions and the development of the disease might be associated with changes in the functioning of VCP. In addition, Vcp protein may be involved in the regulation of mitochondrial function in the cell, and mitochondrial dysfunction also plays an important role in the pathogenesis of PD^26^.

Other key pathological processes in PD are mitochondrial dysfunction^27–31^ and impairment of ubiquitin-dependent proteolysis^27–29,31,32^, which may also be associated with impaired VCP functioning. However, no studies have been reported on the changes in the expression of the *VCP* gene during the development of the early stages of the sporadic form of PD, which is not caused by any mutations and constitutes more than 85% of cases of PD^33,34^.

Therefore, we carried out an analysis of changes in the expression of this gene at the mRNA and protein levels in tissues of mice with a 1-methyl-4-phenyl-1,2,3,6-tetrahydropyridine (MPTP)-induced model of the earliest stages of PD^35,36^, as well as in patients with “predicted” PD and in treated and untreated patients at stages 1 and 2 of the Hoehn–Yahr scale.

## Results

In this study, the analysis of the changes in *Vcp* gene expression was performed using mRNA and protein levels in the brain and peripheral blood of mice with the MPTP-induced models of the earliest stages of PD (Table 1).

**Table 1:** *Vcp* expression changes in the brain and peripheral blood of mice with MPTP-induced PD.

	Cortex				Striatum				Substantia Nigra				Peripheral blood			
	6hPSS	24hPSS	AdvPSS	ESS	6hPSS	24hPSS	AdvPSS	ESS	6hPSS	24hPSS	AdvPSS	ESS	6hPSS	24hPSS	AdvPSS	ESS
mRNA	1.22^a^	**0.19**	**0.48**	**0.44**	0.95	1.24	**0.40**	**0.03**	1.57	**3.23**	**1.53**	**0.40**	0.99	**2.05**	**1.52**	**0.76**
	0.14–3.76^b^	**0.12–0.26**	**0.28–0.67**	**0.13–0.73**	0.46–3.98	0.58–2.54	**0.25–0.59**	**0.02–0.05**	0.57–2.58	**1.63–4.60**	**1.23–1.77**	**0.31–0.51**	0.82–1.09	**1.18–3.33**	**1.38–1.88**	**0.31–0.51**
	0.73^c^	**0.0003**	**0.02**	**0.02**	0.77	0.88	**0.01**	**0.009**	0.75	**0.0009**	**0.02**	**0.01**	0.73	**0.04**	**0.02**	**0.01**
Protein	1.17	1.31	0.31	0.94	0.93	1.37	**0.04**	**3.54**	**4.88**	**0.76**	**2.09**	**2.00**	—	—	—	—
	1.15–1.19	1.27–1.35	0.30–0.32	0.93–0.95	0.91–0.96	1.30–1.48	**0.03–0.05**	**3.45–3.56**	**4.87–5.06**	**0.73–0.84**	**2.06–2.10**	**1.94–2.07**				
	0.21	0.09	0.06	0.12	0.09	0.14	**0.007**	**0.03**	**0.01**	**0.02**	**0.01**	**0.01**				

^a^Median; ^b^25–75 percentiles; ^c^p-value. The data in bold are statistically significant (*p*<0.05). The levels of the transcripts studied in the control group were taken as 1. The data are presented as a fold change (N-fold) in expression compared with the control.

**6hPSS** – MPTP-induced model of the presymptomatic stage of PD with decapitation of mice 6 h after MPTP administration; **24hPSS** – MPTP-induced model of the presymptomatic stage of PD with decapitation of mice 24 h after MPTP administration; **AdvPSS** – MPTP-induced model of the advanced presymptomatic stage of PD with decapitation 2 weeks after MPTP administration; **ESS** – early symptomatic stage of PD with decapitation 2 weeks after MPTP administration.

As can be seen from the presented data (Table 1), the statistically significant changes in the relative mRNA levels of this gene were observed in almost all studied tissues of mice with the exception of the earliest presymptomatic stage of PD with decapitation of mice 6 h after MPTP administration (6hPSS). Note that a decrease in the mRNA levels of this gene relative to the control was observed in all tissues examined at the early symptomatic stage of PD (ESS). It also draws attention to the fact, that the profiles of changes in the relative levels of mRNA of this gene were similar in the substantia nigra and peripheral blood of mice with MPTP-induced PD. Analysis of changes in the Vcp protein level revealed the largest changes in the substantia nigra at all stages studied, and also in the striatum at advanced presymptomatic stage (AdvPSS) and ESS.

Next, we analyzed the changes in the relative mRNA levels of *VCP* and of *SNCA* genes, a declared marker of neurodegeneration, in human samples. The analysis was carried out for treated and untreated patients at the early clinical stages of PD (stages 1 and 2 of the Hoehn–Yahr scale). In addition, analysis of these genes was conducted in the group of neurological controls to assess if the observed changes in the relative levels of transcripts were specific for the pathogenesis of PD. This group included the patients with various neurodegenerative diseases. The results are shown in Table 2.

**Table 2:** Changes in the relative mRNA levels of *VCP*, *SNCA* in the peripheral blood of patients.

Genes	Treated patients with PD	Untreated patients with PD	Neurological control	Patients with “predicted” PD
***SNCA***	**0.18**^a^	**0.25**	**0.14**	**0.54**
	**0.05–0.29**^b^	**0.06–1.22**	**0.10–0.86**	**0.48–0.72**
	**0.001**^c^	**0.004**	**0.004**	**0.03**
***VCP***	1.11	**0.43**	0,97	**0.54**
	0.37–2.06	**0.19–0.95**	0.43–2.25	**0.25–0.64**
	0.61	**0.0003**	0.79	**0.01**

^a^Median; ^b^25–75 percentiles; ^c^p-value. The data in bold are statistically significant (*p* < 0.05). The levels of the transcripts studied in the control group were taken as 1. The data are presented as a fold change (N-fold) in expression compared with the control.

As can be seen from Table 2, there was a statistically significant decrease in the mRNA level of the *SNCA* and *VCP* genes in patients with PD. So, all data obtained for *SNCA* were statistically significant. At the same time, the data obtained for *VCP* were specific only for PD, because no changes were observed in the group of neurological control. It is noteworthy that this decrease in the relative mRNA levels of *VCP* was detected in the group of untreated patients with PD and also in the group of patients with “predicted” PD. In addition, it should be noted that the presence of drug treatment in patients with PD can influence the expression of VCP gene. So, we observe different relative levels of this gene mRNA in the group of treated patients with PD (Median - 1.11) and in the group of untreated patients with PD (Median - 0.43).

## Discussion

Currently, much evidence has accumulated showing that PD has a very long prodromal stage that can last up to 20 years^37,38^. This feature of PD does not allow for direct study of the processes at the earliest stages of pathogenesis in patients with this disease. One of the approaches to investigate early stages of PD is the study of models that reproduce the earliest stages of pathogenesis. In the present study, we used MPTP-induced models of the presymptomatic stages of PD (6hPSS, presymptomatic stage of PD with decapitation of mice 24 h after MPTP administration (24hPSS), and AdvPSS) and ESS. Administration of MPTP allows modelling the death of DAergic neurons in the substantia nigra, the deficiency of dopamine in the striatum, and the manifestation of the classic signs of PD^39,40^. PD models based on injections of MPTP are the ones of the most adequate experimental models of this disease. An important advantage of the use of MPTP is that it selectively penetrates in DAergic neurons because of its high affinity for the dopamine transporter DAT and selectively inhibits complex I of the mitochondrial electron transport chain, causing oxidative stress and impaired calcium homeostasis. These events lead to the degeneration of DAergic neurons by necrosis or apoptosis^41–43^. For the models that were analyzed in this study, it was previously shown that both the death of DAergic neurons and the decrease in the level of dopamine in the striatum are observed even at the earliest time intervals, 6 hours after the introduction of the toxin. At the same time, the first motor impairment was observed only in mice with the MPTP model of ESS^35,36^.

The modeling of PD in mice was supplemented with a study of changes in *VCP* expression levels in humans. This made it possible to identify changes characteristic of the pathogenesis of human PD, and also to better understand the general mechanisms of the functioning of the nervous system.

**Figure 1:**
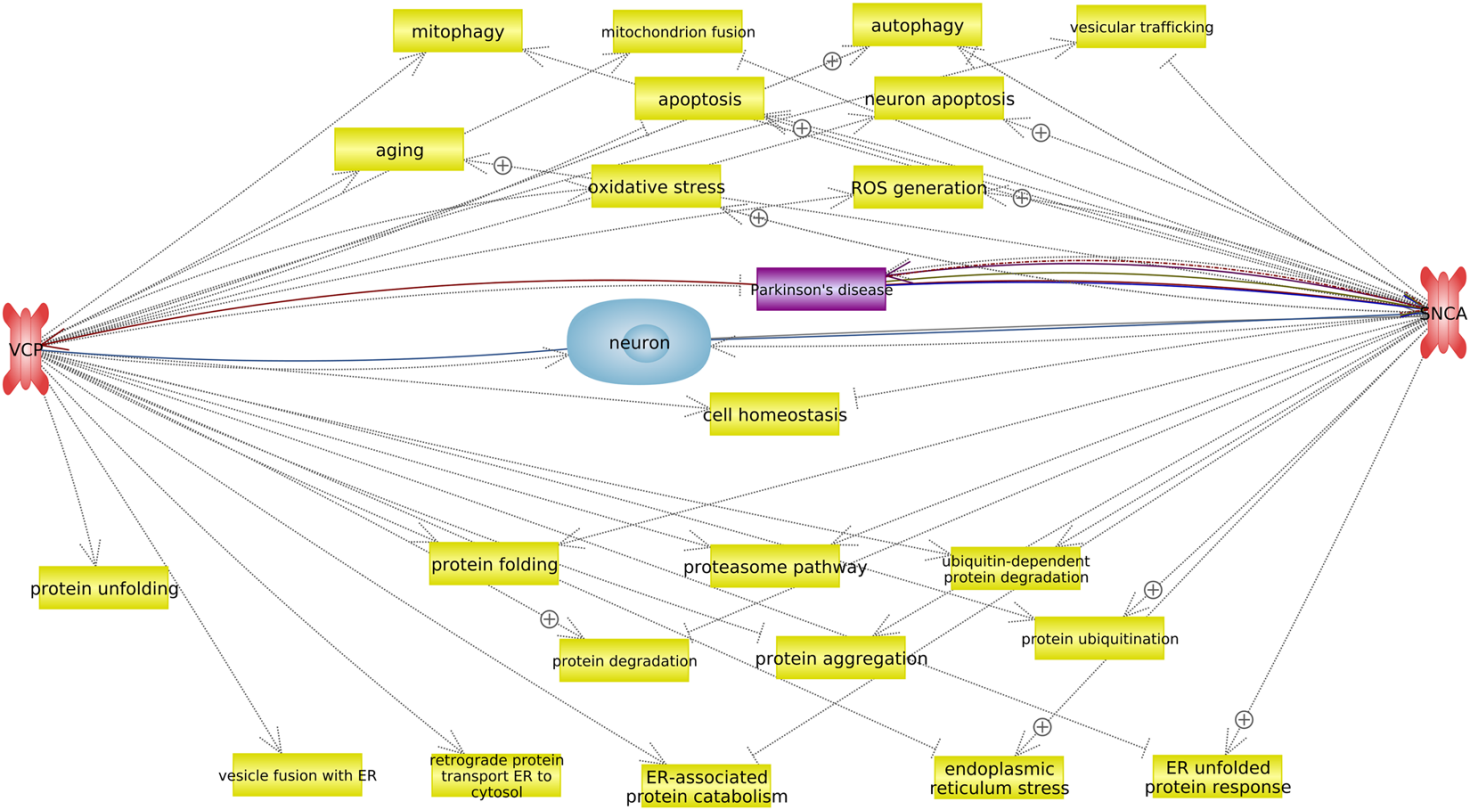
Network demonstrating the main processes involving VCP and SNCA in the pathogenesis of PD. Processes are highlighted in yellow, genes studied are in red, disease (PD) is in purple, and cells (neuron) are in blue. The colored arrows indicate the relationship between the gene and the object: regulatory pathways are in gray, genetic changes are in burgundy, and arrows marked with + indicate positive links (by Pathway Studio v.12.1.0.9).

So, in the first stage of the study, the analysis of the changes in the expression of the *Vcp* gene was carried out at the level of mRNA and protein in the substantia nigra, striatum, cortex, and peripheral blood of mice using four models of the early stages of PD. This gene was selected for analysis because of its role in the pathogenesis of PD. The key processes involving *VCP*, as well as *SNCA*, a well-known marker of neurodegeneration in PD, are represented in the network shown in Fig. 1.

**Figure 2:**
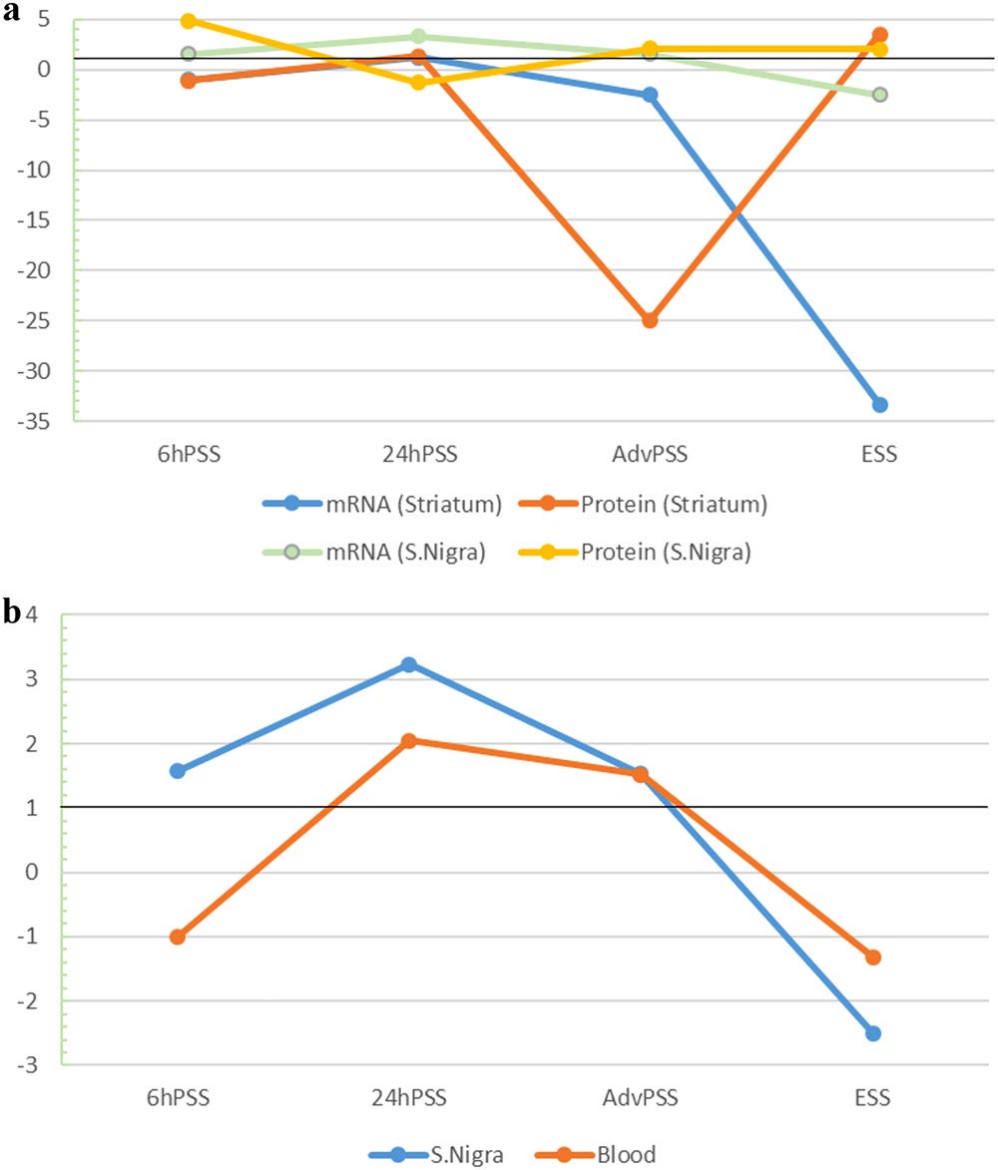
The relative levels of the Vcp gene expression. A The relative levels of mRNA and protein of the Vcp gene in the substantia nigra (S.Nigra) and striatum of mice with MPTP-induced models of PD. B The relative levels of Vcp mRNA in the substantia nigra and striatum of mice with models of PD induced by MPTP. Black line shows levels of Vcp in the control group taken as 1. For clarity of the data presentation, a decrease of expression is presented as “−1/N-fold”.

The most pronounced changes in the expression of *Vcp* at the levels of mRNA and protein were in the substantia nigra and striatum (Table 1 and Fig. 2A).

The predominant increase of gene expression at mRNA and protein levels was observed at the presymptomatic stages in the substantia nigra, which may indicate the development of compensatory mechanisms in the bodies of DAergic neurons, which are mainly located in this part of the brain. Such an increase in expression may be caused by the need to remove aberrant proteins, whose accumulation may occur because of the development of neurodegenerative mechanisms, particularly oxidative stress. This accumulation and aggregation of proteins, as well as the development of oxidative stress, are characteristic features of PD^44^.

At the same time, abrupt falls in the relative levels of mRNA and Vcp protein were observed at AdvPSS in the striatum (Table 1 and Fig. 2A), which indicates an increase of neurodegeneration processes. It was shown that DAergic neuron terminals undergo degeneration earlier than their bodies^45,46^. In this regard, there is an urgent need to maintain protein homeostasis, which can be disrupted because of the development of neurodegenerative processes in the DAergic neuron terminals. It is known that Vcp is actively involved in maintaining protein homeostasis. It appears that nerve cells act to prevent the development of neurodegenerative processes in their terminals. Therefore, a doubling of the relative level of *Vcp* mRNA was observed in the substantia nigra at AdvPSS, which most likely was reflected in the increased protein level at ESS. It is possible that such an increase in the relative level of Vcp protein was associated with an attempt to compensate for its deficiency (Table 1) by transporting it from the substantia nigra to the striatum. This hypothesis is supported by data obtained on a zebrafish model using morpholino oligonucleotides. In that work, on the one hand, the data obtained indicated that the suppression of Vcp expression led to the development of neurodegenerative processes, the pathological accumulation of various proteins, and the blocking of ubiquitin–proteasome protein degradation. On the other hand, it was shown that this phenotype was able to recover to normal through Vcp protein overexpression^9^.

It is also interesting that the profiles of changes in the relative levels of *Vcp* mRNA in peripheral blood and the substantia nigra in mice with MPTP-induced PD matched each other (Fig. 2B and Table 1).

These data are consistent with those on changes in the mRNA levels of a number of genes in mice with MPTP-induced models of PD, obtained by our group previously^47^. It seems that peripheral blood lymphocytes can reflect the processes occurring in the brain, and, in particular, in DAergic neurons^48–50^. These data suggest that peripheral blood lymphocytes can be used in the diagnosistics of early stages of PD, including the presymptomatic stages. Thus, the mRNA levels of genes, including *Vcp*, in peripheral blood can be considered as biomarkers of the neurodegeneration of PD.

In addition, interesting data were obtained on changes in expression in the cortex. Thus, a significant decrease (not less than two times) of the relative levels of *Vcp* mRNA is observed in the cortex of mice with 24hPSS, AdvPSS and ESS. These data indicate that changes in the expression of the *VCP* gene at the very early stages of the pathological process also occur in this structure. Previously, we have already shown the active involvement of the cortex in neurodegeneration processes in mice with early stages of PD^47^.

In the second stage of our study, the analysis of changes in the relative mRNA levels of *VCP* and *SNCA*, a known marker of neurodegeneration, was performed in the peripheral blood of patients with PD. The analysis was carried out in different groups of patients and controls. Two groups of treated and untreated patients with PD (stages 1 and 2 of the Hoehn–Yahr scale) and a group of patients with “predicted” PD were studied. The group of neurological controls was formed to determine whether the changes identified were specific for the pathogenesis of PD. This group of neurological controls was used because the pathogenesis of their diseases was significantly different from that in PD but was directly related to the central nervous system and neurodegenerative processes.

As can be seen from the presented data (Table 2), a statistically significant drop in *VCP* mRNA levels by approximately twofold was seen in the group of untreated patients with PD and in the group of patients with “predicted” PD. Thus, the observed changes were specific for PD, because no changes in the expression of this gene were found in the group of neurological control.

It should be noted that mutations were identified earlier in patients with signs of parkinsonism^17,18^; however, the correlation of changes in *VCP* expression with the sporadic form of PD has not been studied. Our data indicate the important value of the change in mRNA levels of *VCP* at the late preclinical and early clinical stages of PD. The changes we observed in patients with PD were similar to those in the substantia nigra and peripheral blood of our mouse model of ESS of PD (Tables 1 and 2). The data obtained indicate a high specificity of the revealed changes in the expression of *VCP* for the pathogenesis of PD, including the very early stages of the disease.

Changes in *VCP* expression levels in the patients with PD and similar changes in expression of *Vcp* in the substantia nigra and peripheral blood of the experimental mice emphasize their important role in the functioning of the nervous system and the whole organism, including in the early stages of pathogenesis of the disease.

In conclusion, analysis of changes in the expression of *Vcp* gene in the brain regions and peripheral blood of mice with MPTP-induced models of the earliest stages of PD indicates that a change in the expression of this gene may reflect the altered functioning of the nervous system in pathological conditions at the early stages of PD. Analysis of changes in the expression of *Vcp* in the tissues of the brain and peripheral blood of mice with MPTP-induced models of the earliest stages of PD indicates that a change in the expression of this gene is important for the altered functioning of the nervous system in pathological conditions at the early stages of PD. Analysis of changes in the relative mRNA levels of *VCP* in patients with “predicted” PD and in treated and untreated patients (at stages 1 and 2 of the Hoehn–Yahr scale) and also for *Vcp* in the peripheral blood of mice used as models of PD revealed significant and specific decreases in expression levels. These data suggest that a decrease in the relative levels of *VCP* mRNA in patients with PD might serve as a useful biomarker for the development of pathology at the early clinical and preclinical stages of this disease.

## Methods

### MPTP models of PD

Mouse models of the presymptomatic stages of PD were used in this study. Groups included 6hPSS and 24hPSS (mice were decapitated 6 h and 24 h after MPTP administration, respectively), and AdvPSS and ESS of PD. The models were created as described previously^35,36^. In brief, male mice C57BL/6 8–12 weeks old weighing 22–26 g were used in this study. The animals were maintained at 21–23 °C in a 12-h light/dark cycle having free access to food and tap water. The animals were divided into four experimental groups with the s.c. injections of MPTP hydrochloride (Sigma, USA). The control animals received saline only. In the first group, MPTP was injected twice with 2 h intervals between the injections at the individual dose of 12 mg/kg (10 animals in the experiment and 10 animals in the control). In other groups, MPTP was injected four times at the individual dose of 12 mg/kg with a 2-h interval between the injections (30 animals in the experiment and 30 animals in the control). The animals from the first group (AdvPSS) were decapitated two weeks after the MPTP administration. The animals from the other groups were decapitated 6 h (6hPSS), 24 h (24hPSS) and two weeks after the last MPTP administration (ESS). Samples of striatum and cortex (5–7 mg each) and the substantia nigra (1–2 mg) of each animal were taken for the study following decapitation. The tissue pieces were weighed, frozen in liquid nitrogen and kept at -70° C until RNA isolation.

Experimental work with laboratory animals was carried out in accordance with The Guide for the Care of Animals^51^ as approved by the Ethics Committee of the Koltzov Institute of Developmental Biology of the Russian Academy of Sciences. Four control and four experimental groups of C57BL/6 male mice (10 animals in each group) were used for expression analysis. The mice were maintained with free access to food and water with a 12:12 h light:dark regime. Samples of 5–7 mg of striatum and cortex and 1–2 mg of the substantia nigra of each animal were taken for the study following decapitation.

### Patients and comparison groups

Five samples of individuals were analyzed in this study.

During sampling the exclusion criteria for all analyzed samples were the presence in individuals of acute stroke, myocardial infarction, cancer, other history of chronic diseases, or acute infectious diseases.

Inclusion criteria were: for patient samples - the presence of appropriate diagnoses, for the control sample - the absence of any neurological diseases.

In this case, two samples of patients with PD were formed. The first sample included 52 treated and untreated patients newly diagnosed with PD (stages 1 and 2 of the Hoehn–Yahr scale). These included 38 untreated and 14 treated patients with the sporadic form of PD at the time blood was taken. The mean age ±standard deviation (SD) at disease onset was 57.8 ± 7.2 years (range 46–72), and the mean age at enrollment was 58.9 ± 6.9 years (range 47–73). The treated patients received different medications with dopamine receptor agonists (pramipexole at a dose of 1.5 mg/day or piribedil at a dose of 150 mg/day, L-dopa at a dose of 150–200 mg/day, or amantadine at a dose of 300 mg/day) either as monotherapy or in various combinations.

These patients (of Russian ethnic origin residing in Moscow) were diagnosed with PD at the Research Center of Neurology (Moscow) and did not have any family history of PD. They were selected and studied according to the international Movement Disorder Society (MDS) Unified Parkinson’s Disease Rating Scale (UPDRS)^52^ and Hoehn–Yahr scores^53^. The diagnosis of PD was based on the UK PD Society Brain Bank Criteria^54^.

The second sample included nine patients with “predicted” PD. Their mean age at enrollment was 58.3 ± 13 years (range 44–86). These patients (of Russian ethnic origin residing in Novosibirsk) were examined at the Novosibirsk State Medical University. (stages 1 and 2 of the Hoehn–Yahr scale). A sample of 12 individuals with an estimated diagnosis of PD based on an analysis of markers of “primary risk” (hyperechogenicity of the substantia nigra) and secondary (“small”) biomarkers of PD (olfactometry, analysis of coordinated movements of the eyes, head and hands, assessment of motor and non-motor symptoms according to the UPDRS and NMSS scales) was formed in 2016. At this point, blood was taken from them for subsequent expression analysis. A diagnosis of PD was confirmed in nine people by the presence of classic motor symptoms of the disease in 2018. These patients were included into the second sample – patients with “predicted” PD.

All patients were examined for the presence of frequent mutations in all genes responsible for monogenic forms of PD.

The following samples were studied in this work as comparison groups. 23 patients with different neurological disorders including amyotrophic lateral sclerosis (age 56.2 ± 2.1 years), Charcot–Marie–Tooth disease (age 18.4 ± 3.6 years), Wilson’s disease (age 22.8 ± 5.2 years), and cerebellar ataxia (age 34.8 ± 3.8 years) from the same population were studied as neurological controls; 44 neurologically normal age-matched healthy individuals were studied as controls. Individuals of Russian ethnic origin residing in Moscow of both sexes were selected for these groups of patients. All patients and neurologically healthy volunteers were selected at the Research Center of Neurology (Moscow).

The study was conducted in accordance with the World Medical Assembly (WMA) Declaration of Helsinki - Ethical Principles for Medical Research Involving Human Subjects. All blood samples were collected with the informed consent of the investigated subjects. The study was approved by the Ethics Committee of the Research Center of Neurology and Novosibirsk State Medical University.

### Expression analysis

#### RNA isolation

Tissue samples from each animal were homogenized using TRI Reagent (MRC, USA). Further fractionation was carried out with the introduction of chloroform to the brain tissue homogenate in TRI Reagent according to the manufacturer’s recommendations. After fractionation, the aqueous phase containing RNA was taken and ethanol was introduced in a 1:1 ratio. Phenol–chloroform fractionation was used to isolate the protein (see below).

Further isolation of total RNA was carried out using RNeasy Mini Kit (Qiagen, Germany) and Quick-RNA MiniPrep Kit (Zymo Research Corp., USA) according to the manufacturer’s recommendations. RNA concentration was measured using Quant-iT RNA BR Assay Kit and a Qubit 3.0 fluorimeter (Invitrogen, USA). RNA quality was assessed using the Experion RNA HighSens Analysis Kit and the Experion instrument (Bio-Rad, USA).

#### Analysis of changes in relative levels of mRNA

Gene expression changes were analyzed using quantitative reverse transcription polymerase chain reaction with TaqMan probes. Single-stranded cDNAs were synthesized three times using 50 ng of total RNA. The reverse transcription reaction was performed on the T3 Thermocycler amplifier (T3 Thermoblock, “Biometra”, Germany) using RevertAid™ H Minus Reverse Transcriptase kit (Thermo Fisher Scientific, USA) as recommended by the manufacturer and a mixture of random hexamer primers (Thermo Fisher Scientific, USA) and oligo (dT)_18_ primers (Thermo Fisher Scientific, USA) in a ratio of 3:2 respectively.

Primers and probes for qPCR were selected using the Beacon Designer 7.0.2 program and the nucleotide sequences of the human *VCP* and mouse *Vcp* genes. *Bcat2* and *Psmd7* were used as reference genes for analysis of changes in relative mRNA levels in mice, and *AARS* and *PSMD7* were used as reference genes for analysis of changes in relative levels of mRNAs in human samples. The sequences of gene-specific primers and probes are listed in Table 3.

**Table 3:** Sequences of gene-specific primers and probes.

Gene	Nucleotide sequences
*SNCA* NM_000345.3	Probe: 5′-VIC-TGTTCTCTATGTAGGCTCCAA-BHQ2-3′ Forward Primer: 5′-AGCAGGAAAGACAAAAGAGG-3′ Reverse Primer: 5′-TTGCTCTTTGGTCTTCTCAG-3′
*VCP* NM_007126.3	Probe: 5′- VIC-CAACAGTGTGGTGTCCTTGTCC-BHQ2-3′ Forward Primer: 5′-ATCGGTTAATTGTTGATGAA-3′ Reverse Primer: 5′- GTCTCTTCTTTCCTTTCAG-3′′
*AARS* NM_001605.2	Probe: 5′-FAM-TATGTTCACTCGTCTGCCACCATCCCAT-BHQ1-3′ Forward Primer: 5′- CGGCAGCGATTTATAGATTTCTTCAAG-3′ Reverse Primer: 5′- GGTGAGATGGGTCAATTGTGTTCA-3′
*PSMD7* NM_002811.4	Probe: 5′-FAM-GCTTCTGTAGGCAGCCCTAGGTCCTTCGG-BHQ1-3′ Forward Primer: 5′-TGTCCTAATTCCGTATTGGTCATCATTG-3′ Reverse Primer: 5′-CGTGTTCAAATGTTTTCGAGGTTGG-3′
*Vcp* NM_009503.4	Probe: 5′-VIC-CTTTGAACTCCACAGCACGCAT-BHQ2-3′ Forward Primer: 5′-CCATCCGTAAAGGAGATA-3′ Reverse Primer: 5′-GAGCAACAATACAGTAAGG-3′
*Bcat2* NM_001243053.1	Probe: 5′-FAM-CGGATACACTCCAACAGCTCCTGCTTGT-BHQ1-3′ Forward Primer: 5′-TCAACATGGACAGGATGCTACG-3′ Reverse Primer: 5′-CCAGTCTTTGTCTACTTCAATGAGC-3′
*Psmd7* NM_010817.2	Probe: 5′-FAM-AGTCCTAGGTCCTTTGGCTTCACGTCGA-BHQ1-3′ Forward Primer: 5′-CTGCACAAGAATGATATCGCCATC-3′ Reverse Primer: 5′-CTCCACTGAGATGTAGGCTTCG-3′

*Accession numbers in GenBank database. FAM and VIC, fluorescent dyes; BHQ1 and BHQ2, quenchers of fluorescence.

cDNA obtained in the reverse transcription reaction was used as a template for qPCR. This was diluted 50 times in an aqueous solution of tRNA from *Escherichia coli* (0.02 ng/μl)^55^. Real-time qPCR was performed using the StepOnePlus™ System (Applied Biosystems, USA) and PCR reagents (Syntol, Russia). The reaction was repeated three times for each cDNA to correct for differences in sample quality and reverse transcription efficiency.

#### Western blot analysis

Total protein samples from phenol–chloroform fractions collected after the isolation of RNA with TRI Reagent (MRC, USA) were processed according to the manufacturer’s recommendations. Protein concentration was measured using Qubit Protein Assay kit and a Qubit 2.0 fluorometer (Invitrogen, USA).

Gradient polyacrylamide gels Any kD™ Mini-PROTEAN^®^ TGX Stain-Free™ Protein Gels (Bio-Rad Laboratories, USA) were used for separating proteins by electrophoresis. Then, 75-μg aliquots of protein were loaded into each lane of the gels, and the resultant protein bands were transferred to Immun-Blot^®^ Low-Fluorescence Membrane polyvinylidene difluoride (PVDF) membranes (Bio-Rad Laboratories, USA) according to the manufacturer’s recommendations using the Transfer of High Molecular Weight Proteins protocol on the Trans-Blot Turbo Transfer System (Bio-Rad Laboratories, USA) within 60 min. In verification experiments, it was shown that all studied proteins bound to the PVDF membranes following this protocol. The membranes were incubated with primary antibodies and nonfat dry milk as the blocking agent at 4 °C with gentle agitation overnight. The primary antibodies used in the analysis were Vcp (1:4000, Abcam, Great Britain) and Actin B (1:10000, Thermo-Fisher Scientific, USA).

After incubation with the primary antibodies, the membranes were washed and then incubated for 1 h in the presence of the relevant affinity-purified secondary antibodies diluted 1:200,000 (IMTEK, Freiburg, Germany) at room temperature. The membranes were washed again and incubated with the chemiluminescent SuperSignal West Femto Maximum Sensitivity Substrate (ThermoFisher Scientific, USA) for 5 min. Chemiluminescence was quantified using a ChemiDoc MP Imaging System and Image Lab software (Bio-Rad Laboratories, USA). Densitometric analysis was performed using the image analysis, processing, and quantification program GelQuant.NET v.1.82^56^.

### Statistical processing and bioinformatic analysis

Primers and probes for analysis were selected using Beacon Designer 7.0 (Premier Biosoft International, USA) and corresponding sequences from the NCBI database^57^. The specificities of primers and probes were checked using Primer3^58^, Primer-BLAST^59^.

The relative levels (R) of transcripts in the test groups were calculated as R = 2^−ΔΔCt^ ^60^. That in the control group was set as 1. Statistical data processing was performed using Statistica for Windows 8.0^61^ and Microsoft Excel 2016 (Microsoft, USA). The relative levels of gene expression were assessed using the Mann–Whitney nonparametric *U* test. Receiver operator characteristic (ROC) curves, and qPCR efficiency and specificity were obtained using the Real Statistics Resource Pack of MS Excel 2016 (Microsoft, USA). Gene interaction networks were built using Pathway Studio^®^ version 12.1.0.9 (Elsevier, Netherlands)^62,63^.

## Data Availability

All data generated or analysed during this study are included in this published article.
